# (*E*)-(4-Bromo­benzyl­idene)amino cyclo­propane­carboxyl­ate

**DOI:** 10.1107/S1600536812002048

**Published:** 2012-01-21

**Authors:** Xing-Hai Liu, Cheng-Xia Tan, Jian-Quan Weng, Hui-Jun Liu

**Affiliations:** a Key Laboratory of Pesticide Chemistry and Applications, Ministry of Agriculture, Beijing, People’s Republic of China; b College of Chemical Engineering and Materials Science, Zhejiang University of Technology, Hangzhou 310014, People’s Republic of China

## Abstract

In the title compound, C_11_H_10_BrNO_2_, the dihedral angle between the benzene and cyclo­propane ring planes is 49.4 (3)°. The C—C—N—O torsion angle is −175.1 (3)°, which indicates that the C=N double bond is in the *E* configuration.

## Related literature

For details of the synthesis, see: Liu *et al.* (2011*a* ▶). For the KARI (ketol–acid reductoisomerase) activity of related compounds, see: Liu *et al.* (2009*a* ▶,*b* ▶, 2010 ▶, 2011*b* ▶,*c* ▶,*d* ▶). For related structures, see: Liu *et al.* (2011*a* ▶,*c* ▶). 
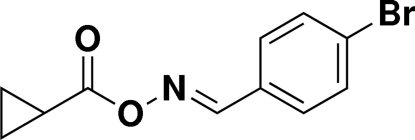


## Experimental

### 

#### Crystal data

C_11_H_10_BrNO_2_
*M* *_r_* = 268.11
Monoclinic, 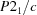

*a* = 13.209 (3) Å
*b* = 13.789 (3) Å
*c* = 5.8714 (12) Å
β = 98.27 (3)°
*V* = 1058.3 (4) Å^3^
*Z* = 4
Mo *K*α radiation
μ = 3.86 mm^−1^
*T* = 113 K
0.16 × 0.14 × 0.12 mm

#### Data collection

Rigaku Saturn diffractometer
Absorption correction: multi-scan (*CrystalClear*; Rigaku/MSC, 2005 ▶) *T*_min_ = 0.577, *T*_max_ = 0.654
5989 measured reflections
1862 independent reflections
1320 reflections with *I* > 2σ(*I*)
*R*_int_ = 0.096

#### Refinement

*R*[*F* ^2^ > 2σ(*F* ^2^)] = 0.038
*wR*(*F* ^2^) = 0.082
*S* = 0.96
1862 reflections
136 parameters
H-atom parameters constrained
Δρ_max_ = 0.67 e Å^−3^
Δρ_min_ = −0.74 e Å^−3^

### 

Data collection: *CrystalClear* (Rigaku/MSC, 2005 ▶); cell refinement: *CrystalClear*; data reduction: *CrystalClear*; program(s) used to solve structure: *SHELXS97* (Sheldrick, 2008 ▶); program(s) used to refine structure: *SHELXL97* (Sheldrick, 2008 ▶); molecular graphics: *SHELXTL* (Sheldrick, 2008 ▶); software used to prepare material for publication: *SHELXTL*.

## Supplementary Material

Crystal structure: contains datablock(s) global, I. DOI: 10.1107/S1600536812002048/ds2156sup1.cif

Structure factors: contains datablock(s) I. DOI: 10.1107/S1600536812002048/ds2156Isup2.hkl

Supplementary material file. DOI: 10.1107/S1600536812002048/ds2156Isup3.cml

Additional supplementary materials:  crystallographic information; 3D view; checkCIF report

## Figures and Tables

**Fig. 1.**
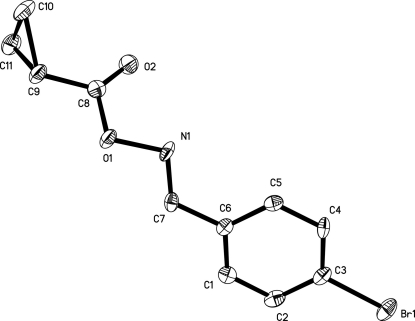
The molecular structure of (I). Displacement ellipsoids are drawn at the 50% probability level and H atoms are shown as small spheres of arbitrary radii.

**Fig. 2.**
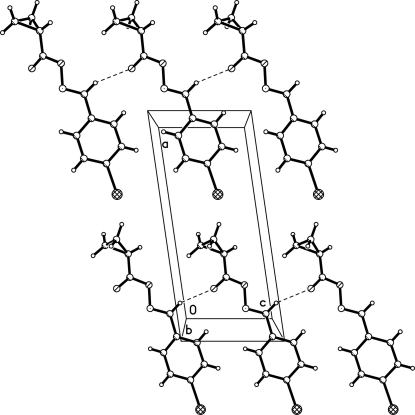
The crystal packing for (I).

## supplementary crystallographic information

### Experimental

To a solution of 4-bromobenzaldehyde oxime (7.50 mmol in 25 ml THF) and 7.5 mmol Et3N, was added the cyclopropanecarbonyl chloride. Then the mixture was vigorously stirred at room temperature for overnight. The corresponding product precipitated immediately. Compound was dissolved in hot alcohol and the resulting solution was allowed to stand in air at room temperature to give single crystal.

### Refinement

All the H atoms were positioned geometrically (C—H = 0.93–0.97 Å) and refined as riding with *U*_iso_(H) = 1.2*U*_eq_(C) or 1.5*U*_eq_(methyl C).

### Crystal data

**Table:**

C_11_H_10_BrNO_2_	*F*(000) = 536
*M**_r_* = 268.11	*D*_x_ = 1.683 Mg m^−^^3^
Monoclinic, *P*2_1_/*c*	Mo *K*α radiation, λ = 0.71073 Å
Hall symbol: -P 2ybc	Cell parameters from 2894 reflections
*a* = 13.209 (3) Å	θ = 2.2–27.5°
*b* = 13.789 (3) Å	µ = 3.86 mm^−^^1^
*c* = 5.8714 (12) Å	*T* = 113 K
β = 98.27 (3)°	Prism, colorless
*V* = 1058.3 (4) Å^3^	0.16 × 0.14 × 0.12 mm
*Z* = 4	

### Data collection

**Table:**

Rigaku Saturn diffractometer	1862 independent reflections
Radiation source: rotating anode	1320 reflections with *I* > 2σ(*I*)
confocal	*R*_int_ = 0.096
ω scans	θ_max_ = 25.0°, θ_min_ = 2.2°
Absorption correction: multi-scan (*CrystalClear*; Rigaku/MSC, 2005)	*h* = −15→14
*T*_min_ = 0.577, *T*_max_ = 0.654	*k* = −15→16
5989 measured reflections	*l* = −6→5

### Refinement

**Table:**

Refinement on *F*^2^	Primary atom site location: structure-invariant direct methods
Least-squares matrix: full	Secondary atom site location: difference Fourier map
*R*[*F*^2^ > 2σ(*F*^2^)] = 0.038	Hydrogen site location: inferred from neighbouring sites
*wR*(*F*^2^) = 0.082	H-atom parameters constrained
*S* = 0.96	*w* = 1/[σ^2^(*F*_o_^2^) + (0.0236*P*)^2^] where *P* = (*F*_o_^2^ + 2*F*_c_^2^)/3
1862 reflections	(Δ/σ)_max_ < 0.001
136 parameters	Δρ_max_ = 0.67 e Å^−^^3^
0 restraints	Δρ_min_ = −0.74 e Å^−^^3^

### Special details

**Table:**

Geometry. All e.s.d.'s (except the e.s.d. in the dihedral angle between two l.s. planes) are estimated using the full covariance matrix. The cell e.s.d.'s are taken into account individually in the estimation of e.s.d.'s in distances, angles and torsion angles; correlations between e.s.d.'s in cell parameters are only used when they are defined by crystal symmetry. An approximate (isotropic) treatment of cell e.s.d.'s is used for estimating e.s.d.'s involving l.s. planes.
Refinement. Refinement of *F*^2^ against ALL reflections. The weighted *R*-factor *wR* and goodness of fit *S* are based on *F*^2^, conventional *R*-factors *R* are based on *F*, with *F* set to zero for negative *F*^2^. The threshold expression of *F*^2^ > σ(*F*^2^) is used only for calculating *R*-factors(gt) *etc*. and is not relevant to the choice of reflections for refinement. *R*-factors based on *F*^2^ are statistically about twice as large as those based on *F*, and *R*- factors based on ALL data will be even larger.

### Fractional atomic coordinates and isotropic or equivalent isotropic displacement parameters (Å^2^)

**Table:**

	*x*	*y*	*z*	*U*_iso_*/*U*_eq_	
Br1	0.63851 (3)	0.63645 (3)	1.06308 (6)	0.02820 (17)	
O1	1.21657 (17)	0.58865 (18)	0.7082 (4)	0.0204 (6)	
O2	1.20566 (19)	0.68074 (19)	0.3851 (4)	0.0246 (6)	
N1	1.1064 (2)	0.5958 (2)	0.6861 (5)	0.0207 (7)	
C1	0.9529 (3)	0.6535 (2)	1.1289 (6)	0.0183 (9)	
H1	1.0055	0.6740	1.2410	0.022*	
C2	0.8530 (3)	0.6600 (2)	1.1709 (6)	0.0206 (9)	
H2	0.8381	0.6849	1.3094	0.025*	
C3	0.7756 (3)	0.6286 (2)	1.0033 (6)	0.0181 (9)	
C4	0.7967 (3)	0.5906 (3)	0.7961 (6)	0.0167 (8)	
H4	0.7441	0.5693	0.6849	0.020*	
C5	0.8972 (3)	0.5849 (3)	0.7583 (6)	0.0177 (8)	
H5	0.9122	0.5592	0.6205	0.021*	
C6	0.9762 (3)	0.6170 (2)	0.9223 (6)	0.0160 (8)	
C7	1.0830 (3)	0.6136 (2)	0.8868 (6)	0.0164 (8)	
H7	1.1344	0.6243	1.0100	0.020*	
C8	1.2549 (3)	0.6312 (3)	0.5280 (6)	0.0187 (9)	
C9	1.3629 (3)	0.6058 (3)	0.5407 (6)	0.0215 (9)	
H9	1.3922	0.5636	0.6676	0.026*	
C10	1.4024 (3)	0.5931 (3)	0.3108 (6)	0.0291 (10)	
H10A	1.4536	0.5436	0.3005	0.035*	
H10B	1.3543	0.6027	0.1714	0.035*	
C11	1.4328 (3)	0.6793 (3)	0.4557 (6)	0.0287 (10)	
H11A	1.4036	0.7414	0.4038	0.034*	
H11B	1.5028	0.6823	0.5328	0.034*	

### Atomic displacement parameters (Å^2^)

**Table:**

	*U*^11^	*U*^22^	*U*^33^	*U*^12^	*U*^13^	*U*^23^
Br1	0.0195 (3)	0.0277 (3)	0.0407 (3)	0.00510 (17)	0.0154 (2)	0.00313 (19)
O1	0.0144 (14)	0.0227 (16)	0.0259 (14)	0.0002 (12)	0.0085 (11)	0.0042 (12)
O2	0.0247 (16)	0.0239 (16)	0.0252 (14)	0.0030 (13)	0.0040 (12)	0.0030 (12)
N1	0.0111 (17)	0.026 (2)	0.0274 (17)	0.0028 (14)	0.0087 (14)	−0.0021 (15)
C1	0.022 (2)	0.012 (2)	0.0204 (19)	0.0001 (16)	0.0028 (16)	0.0003 (15)
C2	0.026 (2)	0.015 (2)	0.0227 (19)	0.0055 (17)	0.0118 (17)	−0.0024 (16)
C3	0.019 (2)	0.011 (2)	0.026 (2)	0.0020 (16)	0.0096 (17)	0.0032 (16)
C4	0.0088 (19)	0.013 (2)	0.028 (2)	0.0003 (16)	0.0012 (15)	−0.0015 (17)
C5	0.021 (2)	0.016 (2)	0.0172 (18)	0.0015 (17)	0.0063 (16)	0.0006 (16)
C6	0.017 (2)	0.012 (2)	0.0206 (19)	0.0013 (15)	0.0054 (16)	0.0020 (15)
C7	0.012 (2)	0.012 (2)	0.025 (2)	0.0015 (15)	0.0013 (16)	0.0026 (15)
C8	0.020 (2)	0.016 (2)	0.0197 (19)	−0.0078 (16)	0.0029 (17)	−0.0065 (17)
C9	0.017 (2)	0.024 (2)	0.0259 (19)	0.0019 (18)	0.0098 (16)	0.0054 (17)
C10	0.023 (2)	0.035 (3)	0.032 (2)	0.001 (2)	0.0157 (18)	−0.004 (2)
C11	0.020 (2)	0.036 (3)	0.032 (2)	−0.009 (2)	0.0099 (18)	0.002 (2)

### Geometric parameters (Å, °)

**Table:**

Br1—C3	1.896 (4)	C5—C6	1.386 (5)
O1—C8	1.369 (4)	C5—H5	0.9300
O1—N1	1.445 (3)	C6—C7	1.456 (5)
O2—C8	1.199 (4)	C7—H7	0.9300
N1—C7	1.285 (4)	C8—C9	1.460 (5)
C1—C2	1.379 (5)	C9—C11	1.504 (5)
C1—C6	1.389 (5)	C9—C10	1.525 (5)
C1—H1	0.9300	C9—H9	0.9800
C2—C3	1.383 (5)	C10—C11	1.483 (5)
C2—H2	0.9300	C10—H10A	0.9700
C3—C4	1.390 (5)	C10—H10B	0.9700
C4—C5	1.379 (4)	C11—H11A	0.9700
C4—H4	0.9300	C11—H11B	0.9700
			
C8—O1—N1	112.4 (3)	C6—C7—H7	119.9
C7—N1—O1	107.6 (3)	O2—C8—O1	124.1 (3)
C2—C1—C6	121.2 (3)	O2—C8—C9	126.9 (3)
C2—C1—H1	119.4	O1—C8—C9	109.0 (3)
C6—C1—H1	119.4	C8—C9—C11	117.6 (3)
C1—C2—C3	118.8 (3)	C8—C9—C10	116.0 (3)
C1—C2—H2	120.6	C11—C9—C10	58.6 (2)
C3—C2—H2	120.6	C8—C9—H9	117.2
C2—C3—C4	121.3 (3)	C11—C9—H9	117.2
C2—C3—Br1	118.6 (3)	C10—C9—H9	117.2
C4—C3—Br1	120.1 (3)	C11—C10—C9	60.0 (2)
C5—C4—C3	118.8 (3)	C11—C10—H10A	117.8
C5—C4—H4	120.6	C9—C10—H10A	117.8
C3—C4—H4	120.6	C11—C10—H10B	117.8
C4—C5—C6	121.0 (3)	C9—C10—H10B	117.8
C4—C5—H5	119.5	H10A—C10—H10B	114.9
C6—C5—H5	119.5	C10—C11—C9	61.4 (2)
C5—C6—C1	118.9 (3)	C10—C11—H11A	117.6
C5—C6—C7	122.6 (3)	C9—C11—H11A	117.6
C1—C6—C7	118.5 (3)	C10—C11—H11B	117.6
N1—C7—C6	120.2 (3)	C9—C11—H11B	117.6
N1—C7—H7	119.9	H11A—C11—H11B	114.7
			
C8—O1—N1—C7	−140.2 (3)	O1—N1—C7—C6	−175.1 (3)
C6—C1—C2—C3	0.5 (5)	C5—C6—C7—N1	11.9 (5)
C1—C2—C3—C4	0.4 (5)	C1—C6—C7—N1	−168.3 (3)
C1—C2—C3—Br1	179.4 (3)	N1—O1—C8—O2	10.1 (5)
C2—C3—C4—C5	−0.4 (5)	N1—O1—C8—C9	−169.7 (3)
Br1—C3—C4—C5	−179.4 (3)	O2—C8—C9—C11	32.6 (5)
C3—C4—C5—C6	−0.4 (5)	O1—C8—C9—C11	−147.6 (3)
C4—C5—C6—C1	1.2 (5)	O2—C8—C9—C10	−33.9 (5)
C4—C5—C6—C7	−179.0 (3)	O1—C8—C9—C10	145.9 (3)
C2—C1—C6—C5	−1.2 (5)	C8—C9—C10—C11	107.8 (4)
C2—C1—C6—C7	179.0 (3)	C8—C9—C11—C10	−105.1 (4)
